# Healthcare costs of patients with chronic obstructive pulmonary disease in Denmark – specialist care versus GP care only

**DOI:** 10.1186/s12913-022-07778-w

**Published:** 2022-03-28

**Authors:** Jesper Lykkegaard, Jesper Bo Nielsen, Maria Munch Storsveen, Dorte Ejg Jarbøl, Jens Søndergaard

**Affiliations:** Department of Public Health, Research Unit of General Practice, University of Southern Denmark, JB Winsløws vej 9A, 5000 Odense, Denmark

**Keywords:** COPD, Cost of illness, Public health, Primary care, Epidemiology

## Abstract

**Background:**

Many patients with chronic obstructive pulmonary disease (COPD) are treated in general practice only and have never received specialist care for COPD. They are seldom included in COPD cost studies but may account for a substantial proportion of the total costs.

**Objective:**

To estimate and specify the total healthcare costs of patients who are treated for COPD in Denmark comparing those who have- and have not had specialist care for COPD.

**Setting:**

Denmark, population 5.7 million citizens.

**Methods:**

Via national registers, we specified the total healthcare costs of all + 30-years-old current users of respiratory pharmaceuticals. We identified the patients with COPD and compared those with at least one episode of pulmonary specialist care to those with GP care only.

**Results:**

Among totally 329,428 users of respiratory drugs, we identified 46,084 with specialist-care- and 68,471 with GP-care-only COPD. GP-care-only accounted for 40% of the two populations’ total healthcare costs. The age- and gender-adjusted coefficient relating the individual total costs specialist-care versus GP-care-only was 2.19. The individual costs ranged widely and overlapped considerably (p25-75: specialist-care €2,175—€12,625, GP-care-only €1,110—€4,350). Hospital treatment accounted for most of the total cost (specialist-care 78%, GP-care-only 62%; coefficient 2.81), pharmaceuticals (specialist-care 16%, GP-care-only 27%; coefficient 1.28), and primary care costs (specialist-care 6%, GP-care-only 11%; coefficient 1.13). The total costs of primary care pulmonary specialists were negligible.

**Conclusion:**

Healthcare policy makers should consider the substantial volume of patients who are treated for COPD in general practice only and do not appear in specialist statistics.

## Background

In developed countries, chronic obstructive pulmonary disease (COPD) is among the diseases that poses the highest economic burdens on the healthcare systems [1]. In Denmark, around 10% of all adults above 30 years of age have COPD [2, 3]. The COPD-specific healthcare costs include costs for hospital admissions mostly for exacerbations, outpatient follow-up visits for COPD, pharmaceuticals for COPD, and COPD-specific services by general practitioners (GPs) and primary care specialists in internal- (including pulmonary) medicine. However, COPD is associated with increased risk of developing almost all other diseases and many COPD-related healthcare costs regard treatment of comorbidities [4–6]. Therefore, estimates of COPD-related costs usually include all excess costs that COPD-patients have compared to otherwise comparable individuals without COPD. Some COPD cost studies are based on small clinically examined or questionnaire surveyed populations [7–9], while the majority are register-based [4–6, 10–15]. A common problem for the register-based studies is that treatment for COPD in general practice is poorly recorded in the registers. As a result, studies fail to account for the costs of patients who are only treated for COPD in general practice and do not appear in specialist statistics. These patients tend to be falsely included in the non-COPD comparator group biasing cost estimates. Knowing their volume and specified costs is important to healthcare planners and economists when allocating resources, budgeting, and organizing the health care systems.

In Denmark, virtually all subjects are listed with a general practice, being the primary caregiver for COPD. Specialist care for COPD requires a referral from the GP [16]. There are negligibly few pulmonary specialists working outside the hospitals. Family medicine is a medical specialty equal to all other most highly ranked medical specialties in terms of duration of training, level of courses and other authorization requirements.

This study aims to estimate and specify the total healthcare costs for patients who are treated for COPD in Denmark comparing those who have- and have not had pulmonary specialist care for COPD.

## Methods

### Design

Register-based nation-wide cohort study.

### Setting and data material

Denmark is a north European country with 5.7 million citizens, each with a unique identification number allowing linkage between national registers. The tax-funded public healthcare system provides almost all primary- and secondary care services free of charge. Outpatients’ pharmaceutical expenses are substantially reimbursed. The Danish National Prescription Registry keeps record of all fillings of prescriptions including the pharmaceutical’s Anatomical Therapeutic Chemical code (ATC) and the price of the purchase [17]. The National Patient Registry (NPR) contains information on all in- and outpatient visits to the few small private- and the many large public hospitals, diagnosis coded using the International Classification of Diseases 10th revision (ICD-10) [18]. The Danish National Health Service Registry contains information on the type and cost of each service provided in primary care, except for few services paid privately or by privately held health insurances [19].

### Population

In the Danish population, we identified all patients aged 30 years and above who redeemed a prescription for a drug for obstructive lung disease (ATC R03) during year 2015 or 2016. This cohort was subsequently split into four mutually exclusive populations (Table 1):

1. Specialist-care: Patients who had had at least one episode of pulmonary specialist care for COPD were identified in NPR as those who during the past five years had at least one episode of in- or outpatient care in any Danish hospital diagnosis coded with ICD-10 code DJ44 or subgroups (COPD) as primary diagnosis or secondary to J13-18 (pneumonia) or J96 (respiratory insufficiency). The Danish Register of COPD uses this validated algorithm [18, 20].
2. GP-care-only: Patients who were treated for COPD in general practice and had had no pulmonary specialist treatment for COPD were identified among the remaining patients as those who on at least two different dates during the years 2015 and 2016 had redeemed a prescription with indication code 379 or 464 (COPD) or on a pharmaceutical with ATC code R03AC18, R03AC19, R03AL02-06, R03BB04-07, or R03DX07 (long-acting muscarinic antagonists (LAMA) in 2016 in Denmark, approved only for treatment of COPD). The Danish Register of Chronic Diseases (RUKS) uses this algorithm to identify COPD outside hospitals [21], except that to increase the specificity of the algorithm opposed to RUKS we required two separate redemptions.
3. GP care LABA (Asthma or COPD): Remaining patients who at least twice had redeemed a prescription on long-acting beta-2-agonist (LABA) alone or in combination with inhaled corticosteroids (ICS/LABA).
4. GP care other R03 (Asthma or COPD): Remaining patients who at least twice had redeemed a prescription on any respiratory drug (ATCR03) being the same or two different drugs.

**Table 1:** Total health care costs in Denmark year 2016 of all patients ≥ 30 years of age treated for obstructive lung disease

Patient population:	Means of identification	No. Pts n (%)	Total costs in 1000 **€** (%)	Costs / pt. yr. in **€**	Coefficient _age & sex adjusted_
Specialist care COPD	COPD diagnosis code < 5 yrs	46,084 (14)	506,909 (34)	11,605	2.19
GP-care-only COPD	Prescription of LAMA or with indication code = COPD	68,471 (21)	344,958 (23)	5,117	1 reference
GP care LABA (Asthma or COPD)	Prescription of LABA or ICS/LABA	116,757 (35)	317,589 (21)	2,736	0.70
GP care other R03 (Asthma or COPD)	Prescription of other ATC R03 drugs	98,116 (30)	339,421 (22)	3,482	0.83

All included patients fulfilled a prescription on a drug for obstructive lung disease (anatomical therapeutic chemical code R03) in year 2015 or 2016

*LAMA* Long-acting muscarinic antagonist, *LABA* Long-acting beta-2-agonist, *ICS* Inhaled corticosteroid, *COPD* Chronic obstructive pulmonary disease

The patient populations are hierarchically exclusive of each other. The coefficients are relative per patient costs compared to GP-care-only COPD

We excluded patients who only redeemed a prescription on ATCR03 on one date during year 2015–2016 or had cystic fibrosis (ICD-10 code DE84* or at least two different redemption dates of prescriptions with indication code 369 or 433 or ATC code R05CB13 (Dornase alfa)).

### Calculation of costs

We included all the secondary care, primary care, and pharmaceutical costs of the patients in 2016 not requiring any direct link to COPD. We did not include the municipalities’ costs of nursing and rehabilitation, and any indirect costs e.g., welfare expenses and income lost due to sick leave. The secondary care costs were calculated based on the patients’ individual data in the NPR using diagnosis-related-group (DRG) tariffs updated on an annual basis. All in- and outpatient hospital services were included. The primary care costs were based on the Danish National Health Service Registry, including services rendered by GPs, primary care specialist physicians, physiotherapists, chiropractors, psychologists, dentists, and foot therapists. The pharmaceutical costs were based on the retail price of each purchased drug including the dispensing costs.

### Analyses

The total and age- and sex-adjusted individual costs were compared across all four groups, but in the following specified analyses, in order to secure a high specificity of the COPD diagnoses in general practice, only population 2) GP-care-only was compared to population 1) specialist-care.

The secondary care costs were divided into the major ICD-10 diagnosis groups based on the primary diagnosis of each episode of care, the primary care costs into the different provider types, and the pharmaceutical costs into the major ATC groups. For each type of cost, the costs per patient year were compared between the two COPD populations. The reported age- and sex-adjusted coefficients indicate the relative difference in annual cost per patient between specialist-care and GP-care-only patients. In the calculations of costs per patient year, each patient only contributed with their time being alive and resident in Denmark.

A sensitivity analysis was conducted expanding the GP-care-only COPD population to also include the patients in population 3) and 4).

All analyses were performed using STATA 14.0 (StataCorp, College Station, TX, USA).

## Results

We identified 329,428 users of respiratory drugs, among those 46,084 with specialist-care- and 68,471 with GP-care-only COPD. The total health care costs for patients with specialist-care or GP-care-only COPD were €852 million corresponding to €7,436 per patient per year. GP-care-only comprised 60% of the COPD population and accounted for 40% of the total costs. In the GP-care-only population 81% were included due to use of LAMA and 19% used another ATCR03 pharmaceutical specifically targeted at COPD (data not shown). Many users of respiratory drugs did not fulfil our GP-care-only criteria and were thus not included in the specified comparing analyses (Table 1).

**Fig. 1:**
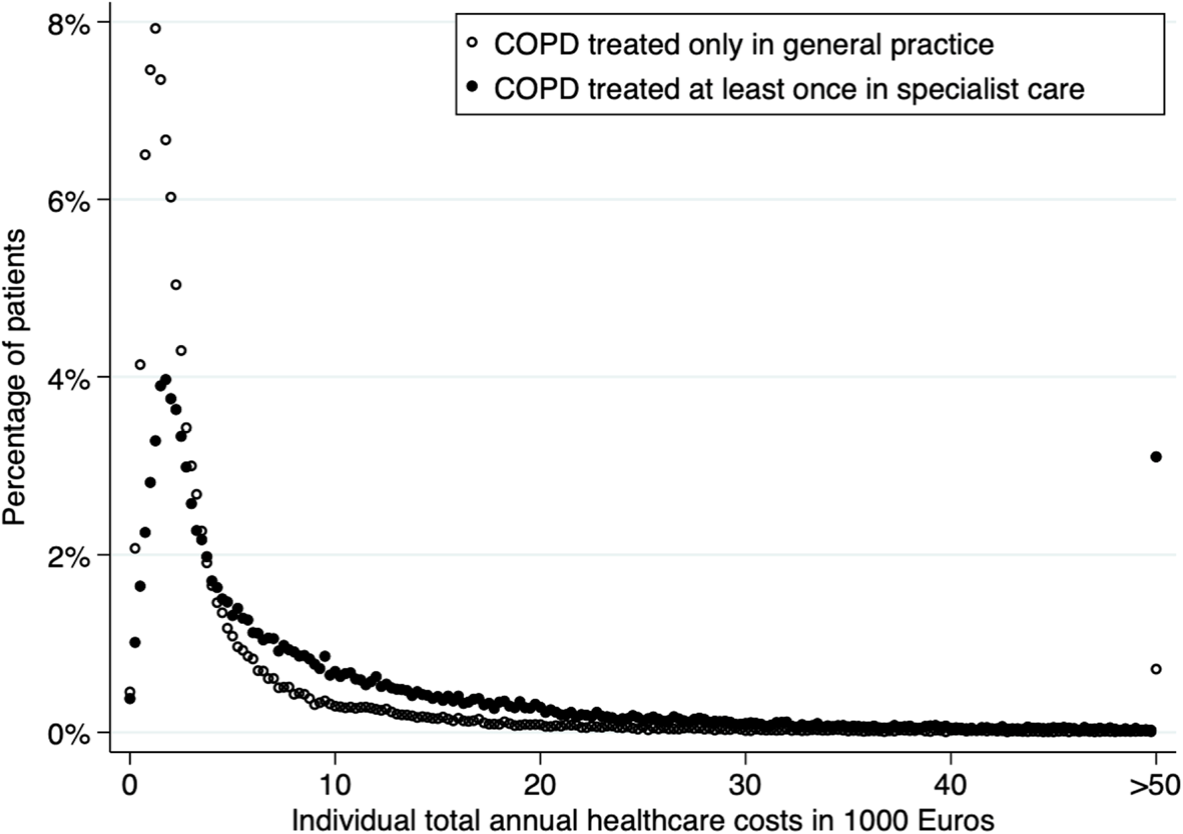
Distribution of patients with COPD in Denmark according to total annual healthcare costs

**Fig. 2:**
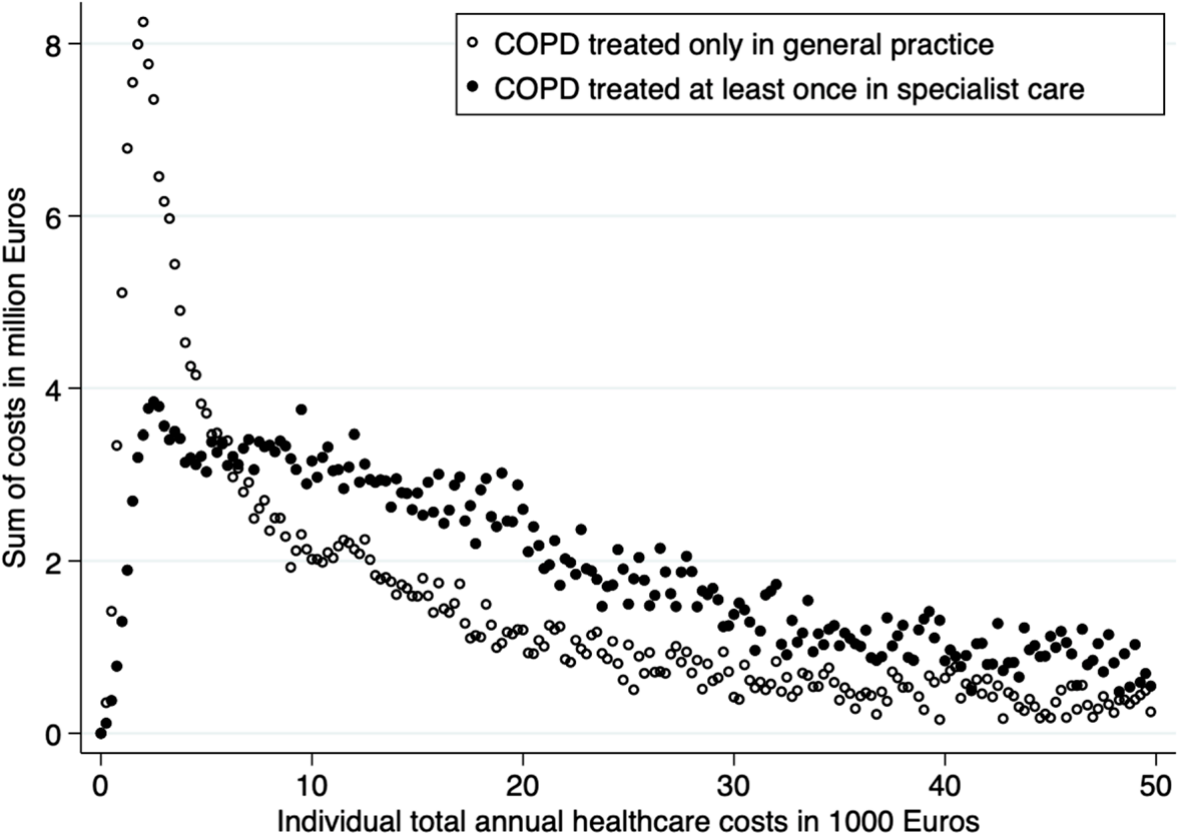
The sum of the two areas under the dots indicate the total healthcare costs for patients with COPD in Denmark. Individual costs > 50,000 € are not shown

The individual annual costs varied widely (p25-50–75: primary-care-only €1,110–€2,100-€4,350 and secondary care €2,175-€5,050-€12,625) (Fig. 1). In both groups, most costs regarded a large proportion of patients with relatively low individual costs (Fig. 2). The cost distribution curves in Fig. 1 were highly skewed and peaked around €1250 per patient year for GP-care-only patients and €1750 for specialist care patients while the mean costs per patient year were €5,117 versus €11,605 (Table 1 and Fig. 1).

Adjusted for differences in sex and age, the total costs per patient year were 2.19 times higher if the patient had been treated in specialist care compared to GP-care-only; costs for hospital services were 2.81 times higher, pharmaceuticals 1.28 times higher, and primary care services 1.13 times higher. In the sensitivity analysis expanding the GP-care-only population to include also population 3) and 4), the adjusted total cost ratio between specialist-care and GP-care-only was 2.58.

The highest total costs were for hospital services, followed by pharmaceuticals, and primary care services. The costs for hospital services were primarily for respiratory diseases, cardiovascular diseases, and cancers. Except for pregnancy- and birth related conditions, the adjusted costs in all ICD-10 diagnosis groups were higher if the patient had been treated in specialist care compared to GP-care-only (Table 2).

**Table 2:** All secondary care costs of patients with COPD in Denmark 2016 specified on the diagnosis registered with each cost

COPD care level five years back:	GP care only (reference)			Specialist care at least once			Comparison
Primary diagnosis (ICD_10_ codes) registered with the costs	Cost total in 1000 **€**	Costs / pt. year in **€**	%	Cost total in 1000 **€**	Costs / pt. year in **€**	%	Coef_Adj_ (95%CI)
Total (any diagnosis)	211,725	3,141	100	395,158	9,047	100	2.81(2.81–2.81)
Respiratory (J*)	23,681	351	11.2	186,975	4,281	47.3	12.0(12.0–12.0)
Cardiovascular (I*)	38,021	564	18.0	40,417	925	10.2	1.58(1.58–1.58)
Factors and contacts (Z*)	31,209	463	14.7	32,275	739	8.2	1.59(1.59–1.59)
Symptoms and findings, NEC (R*)	16,029	238	7.6	24,608	563	6.2	2.31(2.31–2.32)
Neoplasm (C* & D00-D48)	27,700	411	13.1	22,247	509	5.6	1.24(1.24–1.24)
Gastrointestinal (K*)	14,376	213	6.8	16,639	381	4.2	1.76(1.76–1.76)
Infection (A*)	9,106	135	4.3	15,256	349	3.9	2.45(2.45–2.45)
Trauma, outer causes (S*)	10,535	156	5.0	12,170	279	3.1	1.63(1.63–1.63)
Musculoskeletal (M*)	13,875	206	6.6	10,764	246	2.7	1.19(1.19–1.19)
Urology (N00-N51)	6,453	96	3.0	9,559	219	2.4	2.13(2.13–2.13)
Endocrine metabolic (E*)	5,529	82	2.6	7,542	173	1.9	2.00(2.00–2.00)
Neurological (G*)	4,291	64	2.0	5,052	116	1.3	1.87(1.86–1.87)
Eyes and ears (H*)	4,914	73	2.3	4,254	97	1.1	1.23(1.23–1.23)
Blood and immune (D50-D99)	2,120	31	1.0	2,500	57	0.6	1.69(1.69–1.70)
Dermatological (L*)	1,603	24	0.8	2,176	50	0.6	2.10(2.09–2.10)
Psychiatric (F*)	1,293	19	0.6	2,035	47	0.5	2.45(2.45–2.46)
Gynaecology and Mamma (N60-N99)	664	10	0.3	469	11	0.1	1.17(1.17–1.18)
Inborn or genetic (Q*)	200	3	0.1	179	4	0.0	1.61(1.60–1.62)
Pregnancy and birth (O* & P*)	94	1	0.0	19	0	0.0	0.52(0.51–0.53)

*Abbreviations: COPD* Chronic obstructive pulmonary disease, *Coef*_*Adj*_ Sex- and age-adjusted coefficient, *CI* Confidence interval, *NEC* Not elsewhere classified, *Factors and contacts* Factors of significance for health state, and contacts to health care services—including for example check-ups and rehabilitation after disease, and admissions with negative findings ruling out suspicion of e.g., myocardial infarction or cancer

The distribution of pharmaceutical costs on ATC-groups varied little between specialist-care and GP-care-only. More than half of the pharmaceutical costs were for respiratory drugs (Table 3).

**Table 3:** Costs of all pharmaceuticals used by patients treated for COPD in Denmark 2016

COPD care level five years back:	GP care only (reference)			Specialist care at least once			Comparison
Drug target (ATC codes)	Cost total in 1000 **€**	Costs / pt. year in **€**	%	Cost total in 1000 **€**	Costs / pt. year in **€**	%	Coef_Adj_ (95%CI)
All pharmaceuticals, total (A00-V20)	93,208	1,383	100	81,942	1,876	100	1.28(1.28–1.28)
Respiratory organs (R* & V01)	52,768	783	56.6	45,009	1,030	54.9	1.21(1.21–1.22)
Coagulation or blood (B*)	5,795	86	6.2	6,087	139	7.4	1.49(1.49–1.49)
Neurology or psychiatry (N03-99)	6,515	97	7.0	5,824	133	7.1	1.47(1.47–1.47)
Pain, muscles, joints (N00-02 & M01-09^c^)	5,358	79	5.7	5,142	118	6.3	1.47(1.47–1.48)
Diabetes (A10)	4,543	67	4.9	3,808	87	4.6	1.32(1.32–1.32)
Hypertension or heart (C00-09^b^)	4,123	61	4.4	3,523	81	4.3	1.24(1.24–1.24)
Other digestive system (A00-16^d^)	2,602	39	2.8	2,946	67	3.6	1.64(1.63–1.64)
Infection (J* & P*)	2,178	32	2.3	2,669	61	3.3	1.80(1.79–1.80)
Thyroid and not-sex hormones (H*)	1,299	19	1.4	1,408	32	1.7	1.59(1.58–1.59)
Urologic disease (G04)	1,531	23	1.6	1,048	24	1.3	1.00(0.99–1.00)
Hyperlipidaemia (C10)	1,749	26	1.9	1,028	24	1.3	0.91(0.91–0.91)
Eyes and ears (S*)	1,123	17	1.2	785	18	1.0	0.97(0.97–0.97)
Gastric acid (A02)	716	11	0.8	601	14	0.7	1.26(1.25–1.26)
Female organs or hormones (G01-03)	923	14	1.0	528	12	0.6	0.86(0.85–0.86)
Dermatology (D*)	702	10	0.8	525	12	0.6	1.11(1.11–1.12)
Osteoporosis (M05)	530	8	0.6	514	12	0.6	1.37(1.36–1.38)
Neoplasms or immune system (L*)	309	5	0.3	120	3	0.1	0.59(0.59–0.60)
Others	444	7	0.5	377	9	0.5	1.41(1.40–1.41)

*Abbreviations: COPD* chronic obstructive pulmonary disease defined; R03 is the anatomical therapeutical chemical code for drugs targeting obstructive lung diseases; ^b^ excluding C05A; ^c^ excluding M05; ^d^ including C05A and excluding A02 and A10; *Coef*_*Adj*_ Sex- and age-adjusted coefficient, *CI* Confidence interval

Most primary care costs were for services in general practice. Compared to GP-care-only patients, specialist care patients had 2.57 times higher per patient costs for general practitioner home visits in daytime and 3.3 times after hours. Also, specialist care patients had higher costs for all other types of GP services and for physiotherapists, psychologists, and foot therapists, but lower cost for all types of primary care specialist physicians, chiropractors, and dentists. The costs of primary care pulmonary specialists were negligible as they only account for a (not distinguishable) share of the very low total costs of all services provided by primary care specialists in internal medicine (Table 4).

**Table 4:** All-cause primary care costs of patients treated for COPD in Denmark 2016

COPD care level five years back:	GP care only (reference)			Specialist care at least once			Comparison
Primary care service	Cost total in 1000 **€**	Costs / pt. year in **€**	%	Cost total in 1000 **€**	Costs / pt. year in **€**	%	Coef_Adj_ (95%CI)
Primary care total	40,025	594	100	29,808	682	100	1.13(1.13–1.13)
GP total	22,199	329	55.5	18,635	427	62.5	1.26(1.26–1.26)
GP daytime consultations	17,637	262	44.1	12,033	275	40.4	1.04(1.03–1.04)
GP daytime phone calls	1,337	20	3.3	1,325	30	4.4	1.49(1.49–1.50)
GP daytime home visits	924	14	2.3	1,822	42	6.1	2.57(2.56–2.58)
GP daytime Emails	879	13	2.2	949	22	3.2	1.57(1.56–1.57)
GP out of hours consultations	321	5	0.8	291	7	1.0	1.48(1.48–1.49)
GP out of hours phone calls	383	6	1.0	568	13	1.9	2.27(2.26–2.28)
GP out of hours home visits	719	11	1.8	1,647	38	5.5	3.30(3.29–3.31)
Internal medicine specialist	895	13	2.2	364	8	1.2	0.66(0.66–0.66)
Ophthalmologist	3,144	47	7.9	2,021	46	6.8	0.94(0.93–0.94)
Otorhinolaryngologist	1,637	24	4.1	981	22	3.3	0.90(0.90–0.91)
Other specialist physicians	3,181	47	7.9	1,945	45	6.5	0.98(0.97–0.98)
Physiotherapist^*^	4,224	63	10.6	3,220	74	10.8	1.16(1.16–1.16)
Chiropractor^*^	185	3	0.5	77	2	0.3	0.68(0.67–0.68)
Psychologist^*^	214	3	0.5	137	3	0.5	1.15(1.14–1.16)
Dentist^*^	3,694	55	9.2	1,880	43	6.3	0.81(0.81–0.81)
Foot therapist^*^	653	10	1.6	548	13	1.8	1.25(1.24–1.25)

*Abbreviations: COPD* Chronic obstructive pulmonary disease, *GP* General practitioner, *NA* Non-applicable; the ^*^costs are exclusive of substantial patient co-payment; *Coef*_*Adj*_ Sex- and age-adjusted coefficient, *CI* Confidence interval

## Discussion

We found that at least 60% of all patients who are treated for COPD in Denmark have never been treated for COPD in pulmonary specialist care. The average individual total health care cost of a patient who have had specialist care treatment for COPD is between 2,19 and 2,58 times higher than that of a patient who have only been treated for COPD in general practice, and the range of individual costs is very wide and highly overlapping between the specialist care- and GP-care-only populations.

Specialist care patients have higher individual costs than GP-care-only for most types of pharmaceuticals and services in both primary and secondary care. It follows the intentions of the Danish health care system that severe cases are referred to specialist care and though the study lacks clinical characteristics of the patients (spirometry measures, dyspnoea score, and exacerbation frequency) our results, especially the higher costs for GP home visits, indicates a higher overall severity among specialist-care patients than GP-care-only [22]. This difference in case severity between specialist-care and GP-care-only impairs any fair comparison of costs and our findings should not lead to the unsupported conclusion that the current patients with COPD in specialist care could have been treated in general practice at a lower cost. Our intentions when comparing the GP-care-only patients’ consumption of health care resources to that of specialist-care are rather to relate the burden of disease in the two populations and explore any differences in the populations’ specified use of healthcare resources.

As confirmed by our findings, in Denmark almost all COPD treatment in primary care is delivered by the GPs. Shortly after the present study period and among other reasons to save hospital resources, the Danish Regions and the GPs agreed that the GPs should take responsibility for treating a larger proportion of the COPD population and therefore receive a capitation fee (€247 per year) replacing the previous fees for daytime consultations, telephone calls, and E-mails used in this study [23]. Notably, the GP costs in Table four sum to somewhat more than €247 because the daytime consultation costs include fees for laboratory test and other services not covered by the new capitation fee. We found that already before the new agreement, the Danish GPs were responsible for treatment of most patients with COPD, and usually without any involvement of pulmonary specialists.

Prior studies, including two from Denmark, have found that patients with COPD have three to five times higher health care costs compared to age- and gender matched control persons without COPD [4, 5, 7, 8, 10, 15]. These studies primarily sampled COPD patients from secondary care and tended to include the GP-care-only patients in the non-COPD control group. Since GP-care-only patients cost more than non-COPD patients but less than specialist care patients, moving them from the COPD to the non-COPD group will increase the per patient costs in both groups. Thus, the potential bias in cost difference can go either way. However, the estimates of total costs become substantially lower when not including the GP-care-only patients.

Lack of spirometry data lowers the validity of the COPD diagnoses in our study. We used the RUKS’ COPD algorithm that was developed by Sundhedsdatastyrelsen [The Danish Board of Health Data] to identify patients with COPD in the national registries. The algorithm intentionally favours specificity over sensitivity. For specialist care it means that the diagnoses used to define COPD are highly specific, but some hospital treatments of COPD are falsely coded as pneumonia or other not COPD diagnoses leading the patient to be misclassified as GP-care-only or not even included in the study [18]. Also, the specificity of the COPD diagnoses in GP-care-only is high since most of the patients purchased LAMA at least twice in the inclusion period, which is a rather expensive drug specifically indicated for moderate to severe COPD. On the other hand, the sensitivity is low. More than twice the included number of patients redeemed prescriptions on pharmaceuticals for obstructive lung disease but did not fulfil the algorithm. Some of these patients probably have asthma and not COPD but, considering that we only include + 30-year-olds, many of the patients most likely have COPD. A known error in the recording of indication codes probably caused some GP-care-only patients not to be included. Prescription of many respiratory pharmaceuticals are by default coded with the indication codes for asthma or bronchospasm and these default codes have been recorded in the registry even if the prescriber corrected the indication on the prescription. That is probably why the GP-care-only cohort consisted of far more LAMA- than LABA- or shortacting-beta-2-agonist users. Consequently, our comparison analyses include only 114,555 patients while the Danish COPD prevalence studies report around 320,000 Danes to have COPD, some of these though estimated to have undiagnosed COPD or be diagnosed with COPD but not pharmaceutically treated for COPD [2, 3].

Despite its known limitations, we used the RUKS’ algorithm partly to concur with the approach of Danish authorities but mostly to optimise the specificity of the COPD diagnoses, acknowledging that in general practice the COPD diagnoses may be less accurate than in specialist care. Even with this very restrictive approach we identified a large cohort of patients with GP-care-only COPD accounting for much of the total costs. RUKS provides sufficient case severity and accuracy of the COPD diagnoses to rely on the shown differences in resource allocation. For example, that patients with specialist care COPD are more likely than GP-care-only patients to use GPs and psychologists, but less likely to use primary care specialists and dentists. Intuitively, one could mistakenly assume that specialist-care patients would use the GP less often, but this is not the case. The specialist-care patients were identified via episodes of hospital care during the past five years, some of these episodes only lasting few days. However, COPD is a chronic progressive disease and therefore it is reasonable and customary to classify patients based on knowledge of prior rather than only current need for treatment. Inclusion of patients in the specialist-care COPD group based on only brief historic need for hospital treatment may explain why a substantial proportion of the specialist-care patients had lower costs of healthcare than the median of the GP-care-only patients. Also noteworthy, most likely some of the specialist-care classified patients were not directly treated by a fully trained pulmonary specialist. Our assumption that hospital-based treatment for COPD equals pulmonary specialist level is however the usual approach and our specialist care cost estimates are generally consistent with previous findings. A study based on the same Danish registries as this study estimated the total costs of healthcare for patients with COPD in specialist care during the period from 1998 to 2010. Compared to that study (and adjusted to 2016-prices) the present study found somewhat lower individual costs for pharmaceuticals (1794 **€** versus 1950 **€**) but considerably higher costs for primary- (729 **€** versus 564 **€**) and secondary health care (8717 **€** versus 6954 **€**) [10]. Another Danish study estimated the primary- and secondary care costs in 2002 and reported similarly lower costs than our estimates [5]. The differences in individual costs compared to the present study probably reflect the ongoing development of better but more complex and expensive health care services and that the prices of inhalator pharmaceuticals in Denmark have decreased. A Swedish study from 2013 found somewhat higher primary and secondary care COPD health care costs but lower costs of pharmaceuticals [4]. A Spanish study reported lower total annual costs per patient than us (€4,238 versus €7,436) maybe because of more sensitive inclusion criteria, other included type of costs or lower salaries to healthcare professionals in Spain than in Denmark [23]. Generally, comparisons to other COPD cost studies are impaired by differences in populations, health care systems, methods et cetera. However, all studies agree that most health care costs relate to hospitalisations, often with comorbidities.

Our findings are probably generalisable to other health care systems with a strong general practice sector e.g., the UK, the Netherlands, and Scandinavia.

### Strengths and limitations

The cost data from Danish registries used in this study are highly complete and valid [17]. All ATCR03 and with few exceptions all other drugs are on prescription. Based on the individual patient’s purchases in the past 365 days, the national electronic prescription system calculates a partial remuneration when buying a prescribed drug. False records of purchases are unlikely since all parts of the healthcare system use the same synchronised medication platform meaning that all purchases are immediately exposed to the patient and all involved healthcare personnel. Primary and secondary care costs in the study are equal to the actual payments to the GPs and hospitals. However, the study neither covers the healthcare costs of services provided by the Danish municipalities, including nursing, prevention, and rehabilitation, nor the societal costs related to production losses and absence from work.

### *I*mplications

Our findings imply that health care planners and researchers doing population- and cost studies of COPD in countries with a strong general practice should be aware that most patients who are treated for COPD are solely treated for the disease in general practice. These patients appear frequently in the secondary healthcare system but not directly with COPD. They account for a large share of the total health care costs of patients with COPD and should not be overlooked when comparing and/or prioritizing disease-related health care resources.

Future studies should aim to further characterize and differentiate patients with COPD in different parts of the health care systems exploring the wide and overlapping range of individual costs. Healthcare professionals, politicians, and patients think, plan, agree on, and draw pyramids where the few most resource demanding COPD patients are supposed to be treated in specialist care and only milder cases in general practice [23, 24]. However, the substantial overlap of the individual costs of specialist-care and GP-care-only patients in Denmark may reflect the weaknesses of the pyramid mindset rather than the Danish healthcare systems’ inability to fit all COPD patients in the pyramid. As our specification of the costs show, multimorbidity is common among patients with COPD and most likely the main reason for the considerable overlap between the individual cost of patients in specialist- and GP care. It may be that for many patients with COPD even among the overall most resource demanding the generalist expertise and continuity of care provided in general practice is more important than specialist care.

## Data Availability

All data are available from the Danish national registers. https://sundhedsdatastyrelsen.dk/da/english/health_data_and_registers.

## Abbreviations

ATC: Anatomical Therapeutic Chemical
COPD: Chronic obstructive pulmonary disease
DRG: Diagnosis-related-group
GP: General Practitioner
GP-care-only: Patients who are treated for COPD in general practice and had had no specialist care for COPD
ICD-10: International Classification of Diseases 10^th^ revision
ICS: Inhaled corticosteroid
LABA: Long-acting beta-2-agonist
LAMA: Long-acting muscarinic antagonist
NPR: National Patients Registry
RUKS: The Danish registry of chronic diseases
Specialist-care: Patients who have had treatment for COPD in specialist care
